# A novel TJP1-ROS1 fusion in malignant peripheral nerve sheath tumor responding to crizotinib: A case report

**DOI:** 10.1097/MD.0000000000020725

**Published:** 2020-06-26

**Authors:** Juming Li, Lingxiang Liu, Qi Zhang, Yumin Huang, Yihong Zhang, Xiaoyan Gan, Siqin Liu, Zhen Yue, Yongzhong Wei

**Affiliations:** a Department of Orthopedics; b Department of Medical Oncology, the First Affiliated Hospital of Nanjing Medical University, Nanjing; c OrigiMed, Shanghai, China.

**Keywords:** crizotinib, malignant peripheral nerve sheath tumor, next-generation sequencing, neurofibromatosis type 1 D483Tfs∗15 germline mutation, TJP1-ROS1 fusion

## Abstract

**Rationale:**

Malignant peripheral nerve sheath tumor (MPNST) is a rare sarcoma. Owing to the lack of specific histological criteria, immunohistochemical, and molecular diagnostic markers, several differential diagnoses must be considered. Advances in molecular testing can provide significant insights for management of rare tumor.

**Patient concerns:**

The patient was a 50-year-old man with a history of lumpectomy on the right back 30 years ago. He felt a stabbing pain at the right iliac fossa and went to the local hospital.

**Diagnosis:**

By immunohistochemistry, the tumor cells stained positively for S-100 (focal +), CD34 (strong +++) and Ki-67 (20%), and negatively for smooth muscle actin, pan-cytokeratin, neurofilament, pan-cytokeratin-L, GFAP, CD31, STAT6, ERG, myogenin, and MyoD1. Combined with the histopathology and immunohistochemistry results, our initial diagnosis was solitary fibrous tumor (SFT) or MPNST. The tissue biopsy was sent for next-generation sequencing. neurofibromatosis type 1 Q1395Hfs∗22 somatic mutation, neurofibromatosis type 1 D483Tfs∗15 germline mutation, and amplifications of BTK, MDM2, ATF1, BMPR1A, EBHA2, GNA13, PTPN11, RAD52, RPTOR, and SOX9, as well as TJP1-ROS1 fusion, CDKN2A-IL1RAPL2 fusion and CDKN2A/UBAP1 rearrangement were identified. Given that NAB2-STAT6 fusion, a specific biomarker of SFT, was not identified in our patient's tumor, the SFT was excluded by through genetic testing results. Therefore, our finally diagnosis was a MPNST by 2 or more pathologists.

**Interventions and outcomes:**

Subsequently, the patient received crizotinib therapy for 2 months and showed stable disease. However, after crizotinib continued treatment for 4 months, the patient's disease progressed. Soon after, the patient stopped crizotinib treatment and died in home.

**Lessons:**

To our knowledge, this is the first report of the TJP1-ROS1 fusion, which expands the list of gene fusions and highlights new targets for targeted therapy. Also, our case underlines the value of multi-gene panel next-generation sequencing for diagnosis of MPNST.

## 1 Introduction

Malignant peripheral nerve sheath tumors (MPNSTs) are rare spindle cell sarcomas arising from peripheral nerve cells, including Schwann cells.^[1]^ They account for 5% to 10% of all malignant STSs, and out of this, half are associated with neurofibromatosis type 1 (NF1) and less commonly with NF2.^[2,3]^ MPNST can be occurred in any part of the body, especially the trunk and extremities.^[4]^ Because of morphological and immunohistochemical complexity, especially outside the NF1 context, the diagnosis of MPNST remains challenging.

Breakthrough of next generation sequencing (NGS) platforms throughout this decade provides affordable and reliable high-throughput sequencing for evaluation of functional DNA variations in many diseases.^[5]^ Advances in NGS technology have made it possible to identify key gene fusions and identify novel therapeutic targets across different types of tumors.^[6]^ With the development of molecular biology, targeted therapy has achieved remarkable success, which have better therapeutic effects and less adverse reactions.^[7,8]^

In addition, NGS genomic profiling may be of great significance in determining the tumor tissue origin,^[9]^ especially for patients with unclear diagnosis by traditional immunohistochemical detection,^[10]^ as well as morphology and history.^[11]^ At present, tumor gene mutation detection based on NGS technology can achieve satisfactory results in establishing a diagnosis and the corresponding targeted therapy based on driver gene mutations.

Here, a 50-year-old man was finally diagnosed with MPNST according to histopathology, immunohistochemistry and NGS results. This patient harbored a novel TJP1-ROS1 fusion and NF1 germline mutation identified by NGS, whom achieved stable disease (SD) under crizotinib treatment.

## 2 Case report

A 50-year-old man underwent lumpectomy on the right back 30 years ago, and the histopathology results showed neurofibroma. A mass (4 × 4 cm) in the right iliac fossa was found during the patient's physical examination 20 years ago, however, the patient did not receive any treatment. An egg-sized mass at the right ilium was found in April 2018. Due to no obvious discomfort or pain, he did not see a doctor. He felt a stabbing pain at right iliac fossa two months later and went to the local hospital on September 24, 2018. A computerized tomography (CT) scan revealed multiple masses in the right psoas major, iliopsoas and para-piriformis and right hip front, most probably neurofibroma. For further diagnosis and treatment, he was transferred to our hospital on October 20, 2018.

**Figure 1:**
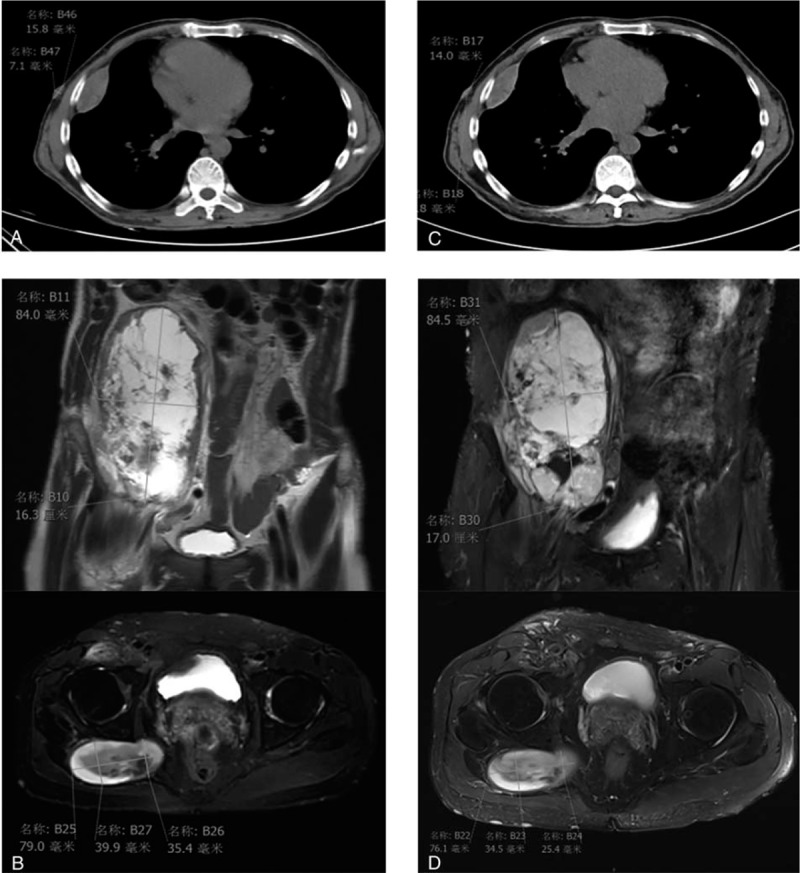
Before crizotinib treatment, CT scans (A), and abdomen MRI (B). After crizotinib treatment for 50 days, CT scans (C), and abdomen MRI (D). CT= computed tomography , MRI = magnetic resonance imaging.

**Figure 2:**
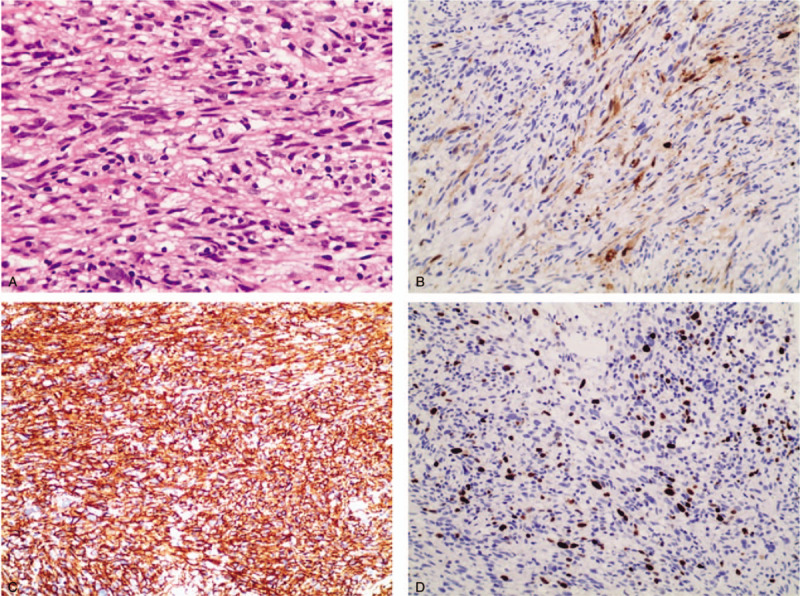
Pathological examinations. (A) hematoxylin eosin staining (magnification, 400×). Immumohistochemical stainings of S-100 (B), CD34 (C), and Ki67 (D) (magnification, 200×).

The patient did not have palpitations, chest distress, low fever, night sweat, or recent body weight changes. Chest CT showed masses in the middle upper lobe of the right lung, and multiple soft tissue nodules in the bilateral parenchyma (Fig. 1A). Combined with medical history, our provisional diagnosis was neurofibroma. There were no obvious abnormalities found in the pelvic X-ray examination. On October 25, 2018, an abdomen magnetic resonance imaging (MRI) (Fig. 1B) revealed an altered marrow signal of the right abdominal cavity and right hip (hypointense on T1, hyperintense on T2, mixed hyperintense on DWI images). There were multiple tiny nodules studded in the abdominal subcutaneous tissue. The mass also involved the enlarged central gland of the prostate, measuring 4.7 × 3.8 cm. Neither obvious abnormal signal in both kidneys nor enlarged lymph nodes around the abdominal aorta were noted. Subsequently, ultrasound-guided fine needle aspiration of the right iliac fossa mass was performed. Pathological examination revealed a spindle cell malignancy (Fig. 2A). By immunohistochemistry, the tumor cells stained positively for S-100 (focal +), CD34 (strong +++), and Ki-67 (20%) (Fig. 2B–D), and negatively for smooth muscle actin, pan-cytokeratin (CK), neurofilament (NF), pan-cytokeratin-L, GFAP, CD31, STAT6, ERG, myogenin, and MyoD1. Combined with the pathological examination, our initial diagnosis was solitary fibrous tumor (SFT) or MPNST.

To make a definite diagnosis and obtain the optimal treatment, the tissue biopsy of patient was prepared for NGS (OrigiMed, Shanghai, China) on November 11, 2018. An NF1 Q1395Hfs∗22 somatic mutation, NF1 D483Tfs∗15 germline mutation, as well as amplifications of BTK, MDM2, ATF1, BMPR1A, EBHA2, GNA13, PTPN11, RAD52, RPTOR, and SOX9 were identified. Importantly, a TJP1-ROS1 fusion (T8; R36), CDKN2A (NM_058195)-IL1RAPL2 fusion (C1; I3), and CDKN2A/UBAP1 rearrangement were also identified by NGS. These genomic alterations were also verified by RNA-sequencing in addition to an EPHB1-MECOM fusion (E1; M2). Since the NAB2-STAT6 fusion is a specific biomarker for SFT, the SFT was excluded by through genetic testing results. Therefore, the patient was finally diagnosed with a MPNST by 2 or more pathologists.

In November 2018, the patient started crizotinib therapy (250 mg, bid). During a reexamination in January 2019, CT scan and MRI showed shrunken local lesion, and the efficacy was evaluated as SD (Fig. 1C-D) according to RECIST1.1 criteria. Unfortunately, during a reexamination in May 2019, CT scan and MRI showed progressive disease. Soon after, the patient stopped crizotinib treatment and died in home in November, 2019. No obvious adverse reactions occurred during crizotinib treatment. This study was approved by the institutional review board of the First Affiliated Hospital of Nanjing Medical University. Written informed consent for the study was obtained from the patient.

## 3 Discussion

Understanding the molecular characteristics of MPNST is critical for its diagnosis and management as it may contribute to the development and application of targeted therapy. In our case the patient developed an egg-sized mass in his right iliac fossa with no initial pain. According to histopathology and immunohistochemistry results, our initial diagnosis was SFT or MPNST. SFT is characterized by the NAB2-STAT6 fusion, which exhibits variable breakpoints and drives STAT6 nuclear expression.^[12]^ Given that NAB2-STAT6 fusion was not identified in our patient's tumor, the SFT was excluded. Therefore, combined with the genetic testing, the patient was finally diagnosed with a MPNST. Our case underlines the value of multi-gene panel NGS for diagnosis of MPNST.

**Figure 3:**
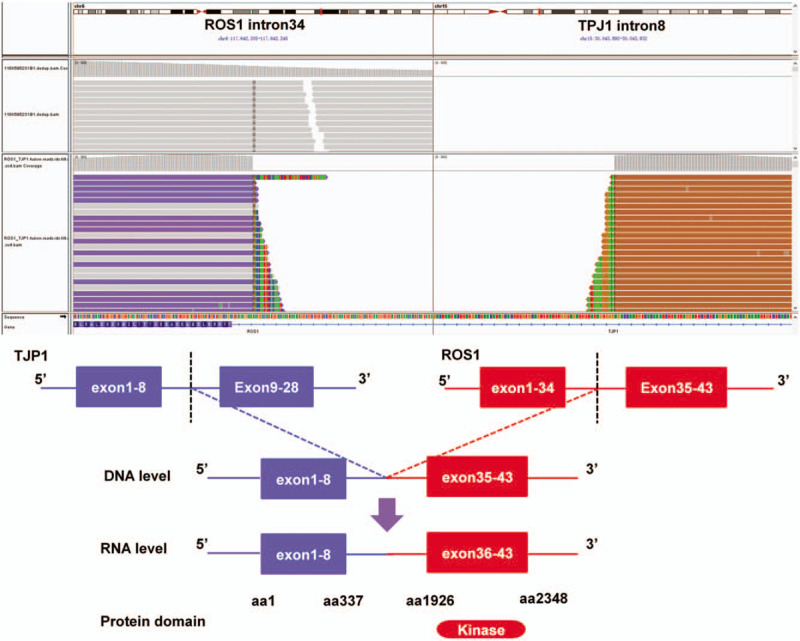
Genomic fusion of exons 1-8 of TJP1 to exons 36-43 of ROS1.

It should be noted that a novel TJP1-ROS1 fusion was identified in the present case. ROS1 is a proto-oncogene located on the long arm of chromosome 6, encoding a receptor tyrosine kinase involved in the regulation of cancer cell growth and differentiation.^[13]^ Fusion products of ROS1 have been observed in a variety of types of cancer, including lung cancer, glioma, hepatic angiosarcoma, gastric adenocarcinoma, and ovarian cancer.^[14]^ This is the first report of a TJP1-ROS1 fusion identified using NGS. The novel TJP1-ROS1 fusion is generated by the fusion of exons 1 to 8 of TJP1 to exons 36 to 43 of ROS1. The predicted TJP1-ROS1 protein product contains 760 amino acids comprising N-terminal amino acids 1 to 337 of TJP1 and C-terminal amino acid 1926 to 2348 of ROS1, retaining the receptor tyrosine kinase domain (Fig. 3).

Crizotinib was the first targeted agent approved by the US Food and Drug Administration for the treatment of ROS1-rearranged non-small cell lung cancer (NSCLC) in 2016, which was based on a phase I expansion cohort crizotinib trial (NCT00585195). The trial demonstrated a median progression-free survival (PFS) of 19.2 months and objective response rate (ORR) of 72% in patients with advanced ROS1-rearranged NSCLC.^[15]^ A phase II trial (NCT01945021) in East Asian patients with ROS1 positive advanced NSCLC reported an ORR of 71.7% and median PFS of 15.9 months in ROS1 fusion patients treated with crizotinib.^[16]^ Another clinical trial conducted in European patients with ROS1-rearranged lung cancer who had undergone documented crizotinib therapy achieved an ORR of 80%, median PFS of 9.1 months, and disease control rate of 86.7%.^[17]^ Considering the patient harbored a ROS1 fusion, he started crizotinib therapy and showed SD after treatment for two months. However, after crizotinib continued treatment for 4 months, the patient's disease progressed. Soon after, the patient stopped crizotinib treatment and died in home.

Other important finding is that NF1 D483Tfs∗15 mutation detected in our case is a heritable germline variation, which may be involved in the relapse of the patient. NF1 is a tumor suppressor gene located on chromosome band 17q11.2. NF1 encodes neurofibromin, which is a member of the GTPase activating protein family. It can activate the GTPase activity of the RAS protein, and then negatively regulate the KRAS pathway. It has been shown that there are more than 500 different NF1 mutations, of which most are unique to the particular kindred.^[18]^ Germline mutation in NF1 gene has been identified in several tumors, including glioma, MPNST, neurofibroma, and so on.^[19–21]^ The germline mutation rate of the NF1 gene (about 1 in 10,000 gametes per cell per generation) is about ten-fold higher than in other inherited disease genes; although the underlying reasons for this new high mutation rate are unclear.^[22]^ This NF1 germline mutation in our case turned 483rd coding aspartic acid into threonine, and terminated the coding at the 15th coding of the new open reading frame, which may cause nonsense mutation-mediated mRNA degradation in the cell, leading to functional deletion of NF1 gene. Given that NF1 D483Tfs∗15 is a heritable germline variation, it may be involved in the relapse of the patient.

To our knowledge, this is the first case of MPNST with a TJP1-ROS1 fusion, raising interesting questions regarding treatment. Also, the NF1 germline mutation in this patient may be related to relapse. In view of the inheritance of NF1 germline mutations, it is necessary to adjust preventive measures for our patient and his family members, and to offer predictive testing to healthy relatives.

## Author contributions

**Conceptualization:** Juming Li, Lingxiang Liu, Yongzhong Wei.

**Data curation:** Qi Zhang, Yumin Huang.

**Investigation:** Juming Li, Lingxiang Liu.

**Methodology:** Yihong Zhang, Xiaoyan Gan, Siqin Liu, Zhen Yue.

**Validation:** Yongzhong Wei.

**Writing – original draft:** Juming Li, Lingxiang Liu.

**Writing – review & editing:** Qi Zhang, Yumin Huang, Yihong Zhang, Xiaoyan Gan, Siqin Liu, Zhen Yue, Yongzhong Wei.
